# Nuclear genome stability in long-term cultivated callus lines of *Fagopyrum tataricum* (L.) Gaertn

**DOI:** 10.1371/journal.pone.0173537

**Published:** 2017-03-09

**Authors:** Alexander Betekhtin, Magdalena Rojek, Joanna Jaskowiak, Anna Milewska-Hendel, Jolanta Kwasniewska, Yulia Kostyukova, Ewa Kurczynska, Natalya Rumyantseva, Robert Hasterok

**Affiliations:** 1 Department of Plant Anatomy and Cytology, Faculty of Biology and Environmental Protection, University of Silesia in Katowice, Katowice, Poland; 2 Department of Cell Biology, Faculty of Biology and Environmental Protection, University of Silesia in Katowice, Katowice, Poland; 3 Kazan Institute of Biochemistry and Biophysics of Kazan Science Centre of the Russian Academy of Sciences, Laboratory of Physiology and Genetics of Plant Cell Cultures, Kazan, Russia; 4 Kazan Federal University, Institute of Fundamental Medicine and Biology, Department of Botany and Plant Physiology, Kazan, Russia; United States Department of Agriculture, UNITED STATES

## Abstract

Long-term cultivated *Fagopyrum tataricum* (L.) Gaertn. (Tartary buckwheat) morphogenic and non-morphogenic callus lines are interesting systems for gaining a better understanding of the mechanisms that are responsible for the genetic stability and instability of a plant tissue culture. In this work, we used histological sections and transmission electron microscopy to identify and describe the morphology of the nuclei of all of the analysed callus lines. We demonstrated that the embryogenic callus cells had prominent round nuclei that did not contain heterochromatin clumps in contrast to the non-morphogenic callus lines, in which we found nuclei that had multiple lobes. Flow cytometry analysis revealed significant differences in the relative DNA content between the analysed calli. All of the analysed morphogenic callus lines had peaks from 2C to 8C as compared to the non-morphogenic callus lines, whose peaks did not reflect any regular DNA content and exceeded 8C and 16C for the line 6p1 and 16C and 32C for the callus line 10p^2^A. The results showed that non-morphogenic calli are of an aneuploid nature. The TUNEL test enabled us to visualise the nuclei that had DNA fragmentation in both the morphogenic and non-morphogenic lines. We revealed significantly higher frequencies of positively labelled nuclei in the non-morphogenic lines than in the morphogenic lines. In the case of the morphogenic lines, the highest observed frequency of TUNEL-positive nuclei was 7.7% for lines 2–3. In the non-morphogenic calli, the highest level of DNA damage (68.5%) was revealed in line 6p1. These results clearly indicate greater genome stability in the morphogenic lines.

## Introduction

*In vitro* propagated plant material can be genetically stable and the clones that are obtained are identical to the parental plant from which the culture was established. However, various, often phenotypic, changes can be observed in the regenerated *in vitro* plants compared to the parental organisms. This phenomenon is known as somaclonal variation. The culprit in such changes may be the rearrangements at the genetic and epigenetic levels that take place during the intermediate stages in a plant tissue culture such as callogenesis and plant regeneration [1, 2]. These include point mutations, structural and numerical chromosomal changes, changes in the gene copy number, alterations in the mobile element activity and changes in DNA methylation [3–6]. Genetic mutations can lead to considerable difficulties in maintaining a callus culture and in plant regeneration and consequently, can prevent or greatly reduce the effectiveness of treatments such as micropropagation or genetic transformation [7].

Somaclonal variation appears to be influenced by the genotype, the explant source, the age of the donor plant and the tissue culture protocol [5]. The precise mechanisms of somaclonal variation are still the subject of debate. A common characteristic of callus cells is their polyploidisation, which has resulted from various processes such as endoreduplication, endomitosis, the fusion of nuclei and chromosomal disorders during anaphase [5]. An increase in the chromosome number during an *in vitro* culture has not only been observed in various plant species, including representatives of the genus *Allium*, such as *A*. *sativum* [8], *A*. *cepa* [9], *A*. *fistulosum* [10], *Solanum tuberosum* [11], *Cucumis sativus* L. [12] and *Agapanthus praecox* [13] but also in the model dicotyledonous plant *Arabidopsis thaliana* [14]. Considerable changes in the global DNA methylation levels as well as methylation at specific sites have been demonstrated for tissue cultures of oil palm [15], grapevine [16] and apple [17]. Cryopreservation, which was applied to *Ribes* genotypes that were not resistant to this process, caused a significant decrease in DNA methylation and, therefore, the activation of previously silenced retrotransposons. A ten-fold increase in the copy number of the Tto1 retrotransposon has also been reported for tobacco plants that had been regenerated from a tissue culture as well as in transgenic plants of this species [18]. A lack of proper DNA methylation contributed to genomic instability, which further hindered the normal development of plants [19].

One good example of the stability of the regeneration potential is the callus of Tartary buckwheat (*Fagopyrum tataricum* (L.) Gaertn.), which has been reported to maintain its ability to undergo somatic embryogenesis and its low chromosome variability through several years of culture [20, 21]. Tartary buckwheat (2n = 16) is a wild buckwheat species that is considered to be a weed in Europe. However, it is widely cultivated in East Asia where it is used for human consumption [22]. Tartary buckwheat is also a source of rutin and other bioflavonoids, which are powerful antioxidants that can be used to treat cardiovascular diseases, inflammation, hypertension, neurological disorders, diabetes and obesity [23]. Buckwheat is the only plant among cereals and pseudocereals whose seeds contain rutin [24]. It is worth mentioning that the rutin content in the groats of Tartary buckwheat is 300-fold more concentrated than that in common buckwheat [25].

The aim of this study was to investigate DNA changes in long-term cultivated calli of *F*. *tataricum* and to compare them to different lines of the morphogenic callus (MC) and the non-morphogenic callus (NC) that were obtained in different years. The extent of the nucleus morphology, relative DNA content and DNA damage were analysed in relation to the callus type and age.

## Material and methods

The seeds of Tartary buckwheat, sample k-17 were obtained from the collection of the N.I. Vavilov Institute of Plant Genetic Resources, Saint Petersburg, Russia (the plants were grown in field conditions from May to September). The lines of calli were obtained from immature embryos of *F*. *tataricum*. Three lines of the morphogenic callus (1–10, K5 and 2–3) and two lines of the non-morphogenic callus (1-10p^2^A and 6p1) were used for the experiments. It should be noted that the non-morphogenic callus 1-10p^2^A was derived from the morphogenic one 1–10, while the non-morphogenic callus 6p1 was derived from the morphogenic line K5. The line 2–3 does not produce any non-morphogenic clone. The calli were cultivated in an incubator at 26°C ± 1 on an RX medium that contained the typical mineral salts of Gamborg’s medium [26], which was supplemented with 2.0 mg/L thiamine-HCl, 1.0 mg/L pyridoxine-HCl, 1.0 mg/L nicotinic acid, 2000 mg/L casein hydrolysate, 2.0 mg/L 2.4-D, 0.5 mg/L indolylacetic acid, 0.5 mg/L naphthylacetic acid, 0.2 mg/L kinetin, 2.5% sucrose and 0.8% agar [20]. The MC and NC callus lines were subcultured every four and three weeks, respectively. The general characteristics of the callus lines, including their origin, type and year of generation are shown in Table 1.

**Table 1:** General characteristics of the analysed callus lines.

Line ID	Type of callus	Origin	Year of generation
1–10	morphogenic	immature embryo	2006
1-10p^2^A	non-morphogenic	line 1–10	2007
K5	morphogenic	immature embryo	2010
6p1	non-morphogenic	line K5	2013
2–3	morphogenic	immature embryo	2012

### Flow cytometry

**Fig 1:**
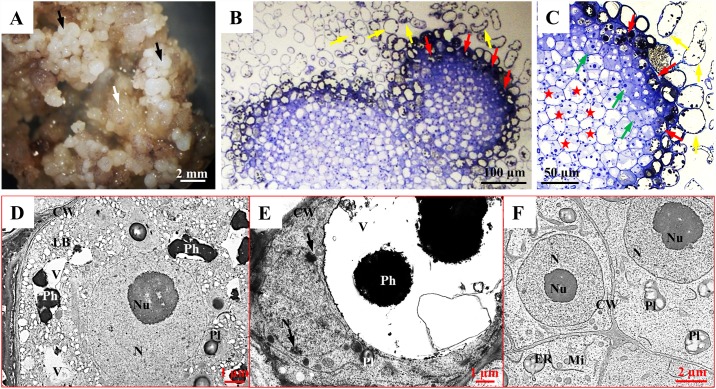
Morphology, histology and TEM of the MC. MC, black arrows—PECC, white arrow–‘soft’ callus (A); histological sections of PECCs (B)–(C); phenolic-containing surficial cells (PC cells) of the PECC, TEM (D)–(E). Arrows indicate heterochromatin clumps; meristematic cells of the PECC (F), TEM. Abbreviations: cell wall (CW), endoplasmic reticulum (ER), nucleus (N), nucleolus (Nu), lipid bodies (LB), mitochondria (Mi), phenolics (Ph), plastid (Pl).

**Fig 2:**
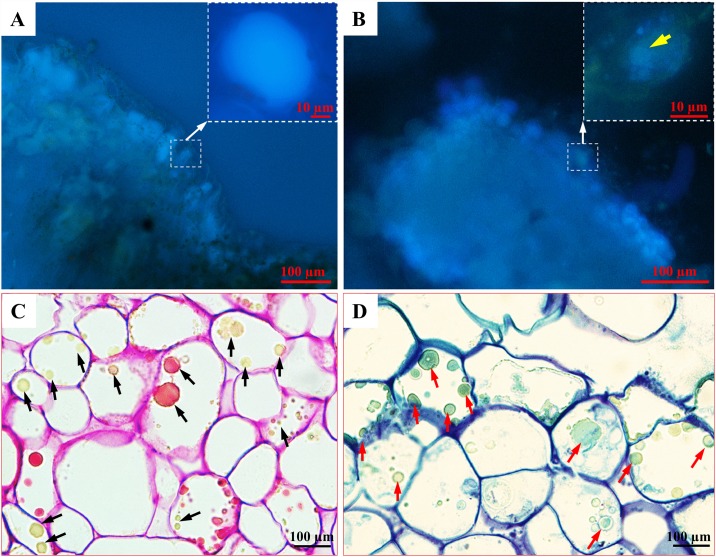
Detection of phenolics in the PECCs. Hand sections through a *C*. *mollis* leaf as a control plant (A) and PECCs in *F*. *tataricum* (B) visualised under UV light, showing the blue autofluorescence of phenolics. The presence of phenolics in a vacuole is marked by a yellow arrow. Sections through the PECCs after PAS reaction (C; orange to yellow colouration of phenolics, black arrows) and stained with TBO in an acetate buffer (D; greenish-blue colouration of phenolics, red arrows).

The relative DNA content of the control plants (Tartary buckwheat plants that had been grown in field conditions from May to September, GPS coordinates: N50.428054, E19.218033) and callus tissues 11 days after the passage onto the fresh medium were analysed using flow cytometry. For each preparation, approximately 0.1 g of young leaf material and each type of callus tissue were used. After mechanical tissue fragmentation, the suspension of nuclei was filtered through a 30-um nylon mesh to remove any large debris and then stained with a staining buffer (Sysmex) that had been supplemented with 1% ß-mercaptoethanol to prevent oxidation of the samples. Samples were incubated for 1–2 minutes and analysed using a CyFlow Space Sysmex flow cytometer with a 365 nm UV LED diode as the light source. Three samples were analysed for each callus type and the flow rate was adjusted to 20–40 nuclei per second. The results are shown on histograms using a logarithmic scale (Figs 1 and 2).

### TUNEL test

The TUNEL test was carried for each line of the calli, which were sampled 2, 11 and 21 days after the passage to the fresh medium. The entire callus tissue and proembryogenic cell complexes (PECCs) were used in the TUNEL reaction. Callus samples were fixed with freshly prepared 4% paraformaldehyde (Fluka) in a 1×PBS (phosphate-buffered saline) buffer for 1 hour at room temperature. Then material was washed 3×5 minutes in PBS. The nuclei preparations were obtained by squashing the callus tissue in PBS. After freezing at -70°C, the preparations were stored for several days at 4°C. Cell permeabilisation was done by incubating the preparations in 0.1% Triton X-100 (Sigma) in 0.1% sodium citrate for 2 minutes at 4°C, after which the preparations were rinsed with PBS. Nuclei that had DNA fragmentation were detected using a TUNEL reaction mixture (*in situ* Cell Death Detection Kit, Fluorescein, Roche). Fifty μl of the TUNEL reaction mixture (enzyme solution: label solution, 1: 9 v/v) was applied to each preparation and incubated for 1 hour at 37°C in the dark in a humid chamber. The positive control preparations consisted of leaf nuclei that had been treated with a DNase solution (1U) for 30 minutes at 37°C in a humid chamber before the TUNEL reaction mixture was applied. The negative control consisted of a mixture without the enzyme. Preparations were rinsed 3× with PBS and stained with DAPI (2 μg/mL), air dried and then mounted in Citifluor.

The preparations were examined with a wide-field AxioImager.Z.2 epifluorescence microscope (Zeiss) equipped with filters for FITC and DAPI. The frequency of the labelled cells was estimated based on 2000 cells that were analysed on three slides for each callus type. The significance of differences between different calli was assessed using the Student's t-test with P < 0.05 taken as indicating significance.

### Light microscopy and transmission electron microscopy

For transmission electron microscopy (TEM), callus pieces (1–2 mm^2^) were fixed in 2.5% glutaraldehyde in a 0.1M phosphate buffer (pH 7.2), washed in the same buffer and then post-fixed in 1% OsO_4_ diluted with the same buffer solution to which sucrose was added (25 mg/ml) for 3 h at room temperature. Tissues were dehydrated through a graded ethanol series, 100% acetone and 100% propylene oxide and embedded in Epon-812 epoxy wax. Sections (70–100 nm thick) were cut on an LKB 8800 (“LKB”, Sweden) ultramicrotome, collected onto nickel grids and stained with 4% uranyl acetate for 20 min and Reynolds lead citrate for 10 min [27]. Observations were made using a Jeol 1200 SX transmission electron microscope (Jeol, Japan). For the histological analysis, semithin sections (3 μm thick) were cut on an Ultra Cut E (Reichert-Jung, Austria) ultramicrotome and stained with 0.05% toluidine blue [28, 29]. The stained sections were examined under a Jenamed microscope (Carl Zeiss, Germany) and recorded digitally using an AxioCam MRc5 camera with AxioVision Rel.4.6 software. In order to confirm that the substances that had been stained with TBO were indeed phenolics, additional reactions were performed. Fresh samples of the *F*. *tataricum* callus lines and *Crepis mollis* were cut with a razor blade, incubated in a drop of demineralised water on a microscopic slide under a coverslip and their autofluorescence was analysed under a wide-field Eclipse Ni-U epifluorescence microscope equipped with a DS-Fi1-U3 digital camera and dedicated software (Nikon, Tokyo, Japan). Sections were exposed to UV light (excitation 365 nm; [30]). *C*. *mollis* leaves were used as the reference sample because of the high content of phenolic compounds [31].

Samples were embedded in LR White resin and cut into 2.5 μm sections [32], then collected onto poly-L-lysine coated microscopic slides and stained using: (a) PAS reaction (periodic acid-Shiff stain; phenolic compounds were visualised with the colour orange; [33]); (b) Toluidine Blue O in 0.1 M acetate buffer at pH = 4.4 (phenolic substances were indicated by greenish-blue to green coloration; [34]).

## Results and discussion

**Fig 3:**
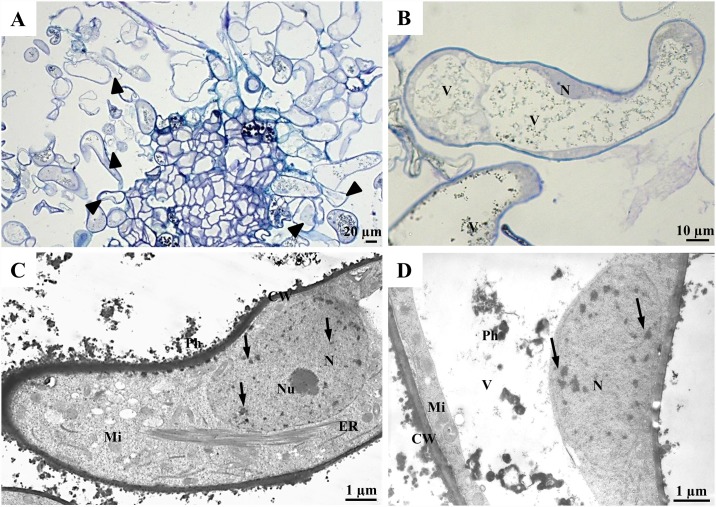
Cells of the ‘soft callus’ in the MC. Histological sections (A)–(B); TEM (C)–(D), single arrows indicate heterochromatin clumps and arrowheads indicate the detachment of plasmalemma from a cell wall.

**Fig 4:**
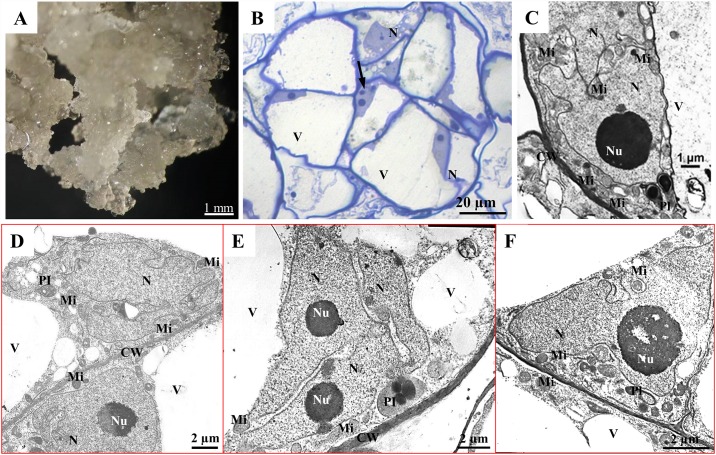
Non-morphogenic callus of Tartary buckwheat. Morphology (A), histological section, arrow shows a cell with numerous nucleoli (B); TEM (C)–(F).

The MC consists of PECCs (or in other words pro-embryogenic masses (PEMs)) and a ‘soft’ callus (Figs 1A–1C, 2B–2D, 3A and 3B). During the cyclical callus development, the mature PECCs disintegrate, thereby giving rise to young PECCs and ‘soft’ callus cells (Fig 3A and 3B) [35, 36]. Non-morphogenic callus lines contain only parenchymatous cells (Fig 4A and 4B) [20, 35]. The PECCs of a Tartary buckwheat callus are structures that are formed by various types of cells (Fig 1B and 1C). The surface of PECCs is covered by cells that accumulate phenolics in their vacuoles, i.e. phenolic-containing cells (PC cells) (Fig 1B and 1C, red arrows), which were manifested by TBO staining (Fig 2D). Interestingly, PC cells separate from the PECCs during the culture (Fig 1B and 1C). When the vacuole of a PC cell is poorly filled by phenolics, the nucleus of such a cell is round with a prominent nucleolus but no heterochromatin clumps (Fig 1D). When a PC cell becomes senescent, its vacuole has significant phenolic depositions (Fig 1E). The nucleus in a senescent cell is flat; the nucleolus, as a rule, disappears and the nucleoplasm has many heterochromatin clumps (Fig 1E). The death of PC cells appears to be very quick—the disruption of the tonoplast releases phenolics from the vacuole and leads to the release of the phenolics. The dead cells are larger than live PC cells (Fig 1B and 1C, yellow arrow). Layers of poorly vacuolised meristematic-like cells that have a dense cytoplasm and round nuclei are located below the PC cells (Fig 1B and 1C, green arrows). Subsurficial meristematic cells are the source of embryogenically determined cells, from which embryoids or new PECCs arise (Fig 1F). Parenchymatous cells that have large starch grains in their plastids constitute the central and most prominent part of PECCs (Fig 1B and 1C, red asterisks).

The presence of phenolics in the surface cells of PECCs was also confirmed by other, more specific methods (Fig 2A–2D). Autofluorescence of the phenolic compounds in the calli cells was compared to *C*. *mollis*, which is known to be a species that contains significant amounts of phenolic compounds [31]. In the Tatary buckwheat callus, at least two layers of surface cells contain phenolics (Fig 1B). The presence of phenolics in the vacuole was also documented (Fig 2B, insertion, yellow arrow). It appeared that the chemical character of the PECC phenolics may be similar to that found in *C*. *mollis* based on their similar autofluorescence hue (Fig 2A and 2B). It was demonstrated earlier that a suspension culture of *F*. *tataricum* contained phenolics such as rutin, quercitrin, epicatechin, ferulic and *p*-coumaric acids [37]. Phenolics deposited in the vacuole may be indicators of stress conditions [38], which is a normal status of cells during an *in vitro* culture [39]. Detection of phenolics using the PAS reaction in peach palm (*Bactris gasipaes*) an *in vitro* culture was obtained [33]. It should be noted that these authors found the phenolics in both the cytoplasm and the adjacent cell wall during the process of their differentiating into somatic embryos. Staining with TBO at a low pH buffer gave the same results as in the case of an *in vivo* analysis of *Eucalyptus festigata* roots [34], which showed a greenish colouration that indicated the presence of phenolic compounds in the calli cell vacuoles.

The ‘soft’ callus is formed during the loosening of the PECCs and primarily consists of elongated cells that are highly vacuolised (Fig 3A and 3B). A drastic difference between the ‘soft’ callus and PECCs was observed. The ‘soft’ callus cells also differ from the NC, since signs of degradation appear in the majority of its cells judging by the detachment of the plasmalemma from the cell wall (Fig 3A). In contrast to the NC, the cells of the ‘soft’ callus probably do not divide. They have a senescent phenotype of nucleus with numerous heterochromatin clumps (Fig 3C and 3D). In the MCs, the ‘soft’ callus has the function of nurse tissue that supports the growth of the PECCs by providing sugars, proteins and other conditioning factors, which are secreted into the culture medium during the loosening and disintegration of the PECCs. The cells of the ‘soft’ callus are metabolically active, but do not divide, thus postponing death through senescence.

The NCs, which are formed exclusively from parenchymous type cells (Fig 4A and 4B), differed from the MCs because of their friable structure, high growth rate and complete loss of the capacity for morphogenesis [21]. In the NC cells, the central vacuole occupies most of the cell and the nucleus has a near-wall position (Fig 4B). There are multinucleated cells quite often; there may be a few nucleoli in one nucleus (Fig 4B). A characteristic feature of the nuclei in the NC is the presence of numerous deep lobes (Fig 4C–4F). In some cases, the lobes are separated so much that it is difficult to identify what is seen on the TEM electronograms—the lobes of one nucleus or two separate nuclei in one cell (Fig 4F). The rough endoplasmic reticulum (ER) has short cisterns and the mitochondria are small and round. The plastids are mostly cup-shaped and have starch grains. In plants that are not exposed to stress *in vivo*, lobed nuclei have been described in the nectary cells of *Padus racemose* [40], in the cells of storage tissues of *Ricinus communis* [40], in the stinging cells of *Urtica dioica* [40], in the root hairs of *Raphanus sativus* [41] and in the tomato pericarp [42]. *In vitro*, lobed or irregular-shaped nuclei have been observed in the non-morphogenic calli of *Beta vulgaris* [43], *Plantago asiatica* [44] and *Allium fistulosum* [10]. The lobed shape of the nucleus appears to be due to the increased amount of nuclear DNA in the differentiated, non-dividing cells (as a result of endopolyploidy) and also in the polyploid dividing callus cells. A correlation between an increased DNA content in the differentiated cells of the tomato pericarp and a change in their shape from round to lobed was found [42]. An increase in cell ploidy has been shown to be accompanied by an increased depth of the nuclear lobes as well as the nucleus area. The prevalence of high-level polyploid cells apparently explains the presence of lobed nuclei in the NC cells of Tartary buckwheat as well as in the NCs of the other above-mentioned species. Nuclear and cytoplasmic volumes are somehow related to each other; this phenomenon is referred to as the karyoplasmic ratio [45] or nuclear/cytoplasmic ratio [46]. It can be assumed that an increase in the surface area of the nuclei in polyploid cells activates the exchange of proteins and ribonucleoproteins between the nucleus and the cytoplasm. An interesting observation was the accumulation of mitochondria in the invaginations of the nuclei (Fig 4C and 4F), apparently in those cell sites where there was the greatest need for ATP; a similar mitochondrial localisation was observed in the cells of the tomato pericarp, which had nuclei with large lobes [42].

**Fig 5:**
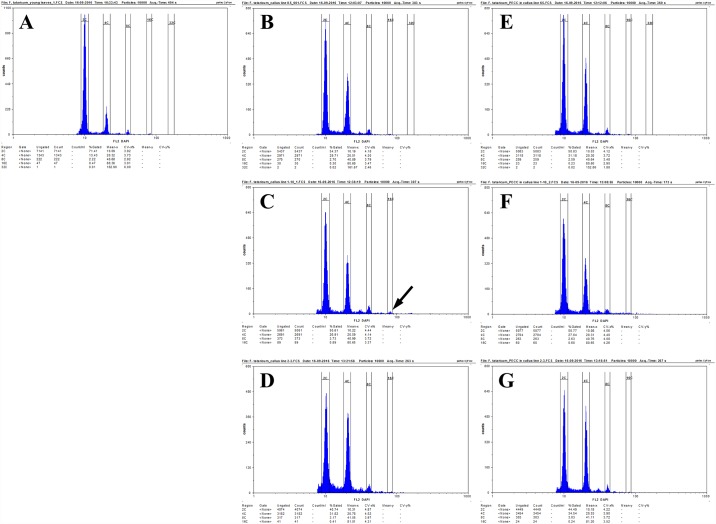
**Flow cytometer histograms showing the relative DNA content in the leaves of the control plants (A) and in the MC.** ‘soft callus’ + PECC for the line K5 (B), 1–10 (C), 2–3 (D) and PECCs without the ‘soft’ callus isolated from the line K5 (E), 1–10 (F) and 2–3 (G).

**Fig 6:**
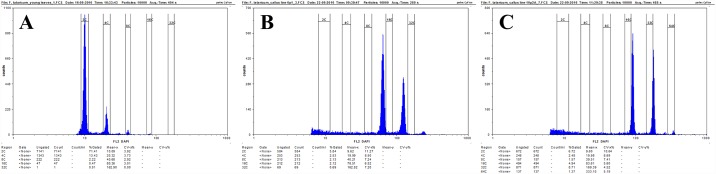
Flow cytometer histograms showing the relative DNA content. Leaves of the control plants (A), NC line 6p1 (B) and NC line 10p^2^A (C).

The cytogenetic and genetic stability of the callus is one of the key prerequisites for the efficient clonal propagation in an *in vitro* plant tissue culture. Long-term callus cultures often result in an accumulation of genetic changes, including polyploidisation. Such changes can lead to the regeneration of polyploids, chimeric plants or even to the loss of morphogenic potential [2, 47, 48]. The calli of Tartary buckwheat that were obtained from immature embryos differed significantly from the non-morphogenic clones in terms of their morphogenetic potential, the types and structure of the cells and biochemical properties [21]. Our flow cytometry analyses revealed significant differences in the relative DNA content patterns between the distinct callus lines of *F*. *tataricum*. The MC lines were primarily characterised by the presence of nuclei that had a relative DNA content at 2C and 4C as well as small peaks at 8C (Fig 5A–5C). In the case of lines 1–10, a small peak that indicated the presence of nuclei with 16C was also detected (Fig 5 –black arrow). The presence of a large population of nuclei with a 4C DNA content and a small population with an 8C and 16C DNA content in the MC may indicate intensive DNA replication before cell division or may be attributed to the occurrence of the polyploidisation cycles that are connected to the endoreduplication processes. The flow cytometry analysis of the PECCs in the morphogenic calli revealed that they had a similar ploidy pattern to the whole callus (PECCs plus the ‘soft’ callus), thus indicating that the MC has a very stable nature in a prolonged culture with most of its cells being diploid (Fig 5E–5G). In contrast, in the NC lines 6p1 and 10p^2^A, the DNA content was significantly different (Fig 6). In these lines, two clearly visible peaks were observed. In the case of the 6p1 callus line, the peaks were gated between a gain for 8C and 16C and a gain for 16C and 32C (Fig 6B). On the other hand, the peaks in the callus line 10p^2^A exceeded 16C and 32C of the DNA content (Fig 6C). These abnormal localisations of the peaks may indicate some changes in chromosome numbers, for example, aneuploidy that was caused by a not purely geometrical progression in the multiplication of the nuclear DNA content that was caused by the fact that subsequent polyploidisation cycles can occur along with the loss of some chromosomes.

The occurrence of populations of nuclei at such diverse DNA ploidy levels indicates the highly unstable genetic nature of the NC lines. These changes can lead to the loss of the regenerative capacity of the callus. In an embryogenic cell suspension of different cultivars of *Musa*, changes in the ploidy level clearly affected the morphogenetic potential. Non-regenerable Grande Naine and Williams suspension cultures were characterised by the presence of highly polyploid cells. In contrast, the regeneration of young plants was observed in the Three Hand Planty cultivar cell culture, which had a ploidy similar to the triploid parental plants [48]. The gradual loss of the ability to regenerate was also a consequence of polyploidisation in cucumber callus cultures, where the plants that regenerated from the callus were mostly diploids (57%) while tetraploids, octoploids and mixoploids contributed only 18%, 4% and 21%, respectively [49]. Changes in the ploidy level during callus cultures have also been reported for *A*. *thaliana* [50], *Oryza sativa* [51], *Hyoscyamus muticus* [52] and *A*. *fistulosum* [10]. The reason for the polyploidisation in the MC callus lines of *F*. *tataricum* could be due to the polysomatic nature of this plant. The degree of polysomaty can be different in juvenile and adult tissues. However, in *Cucumis sativus*, polysomaty was already present in the radicle and hypocotyl of the ungerminated seeds [53]. This indicates that endopolyploidisation had taken place in a number of the cells of these organs at the early stages of tissue differentiation during embryo development. It should be noted that *A*. *thaliana* is also a polysomatic plant that has mixoploid (2C-32C) somatic tissues and meristematic tissues that consist only of diploid cells [54]. An increase in the ploidy level might lead to a decrease in or even total loss of the regeneration potential [55] or to the regeneration of polyploid plants as, for example, in *A*. *thaliana* [14] and *Vitis vinifera* [56]. In contrast to the NC lines, all of the MC lines retained their cytogenetic stability even after several years of cultivation and most of their nuclei had a 2C DNA content. Earlier, such a high cytogenetic stability in a callus culture and regenerants was also reported for *Crepis tectorum* [57], *Pinus taeda* [58], *Ruscus hypophyllum* [59] and *Larix × eurolepis* [60].

**Fig 7:**
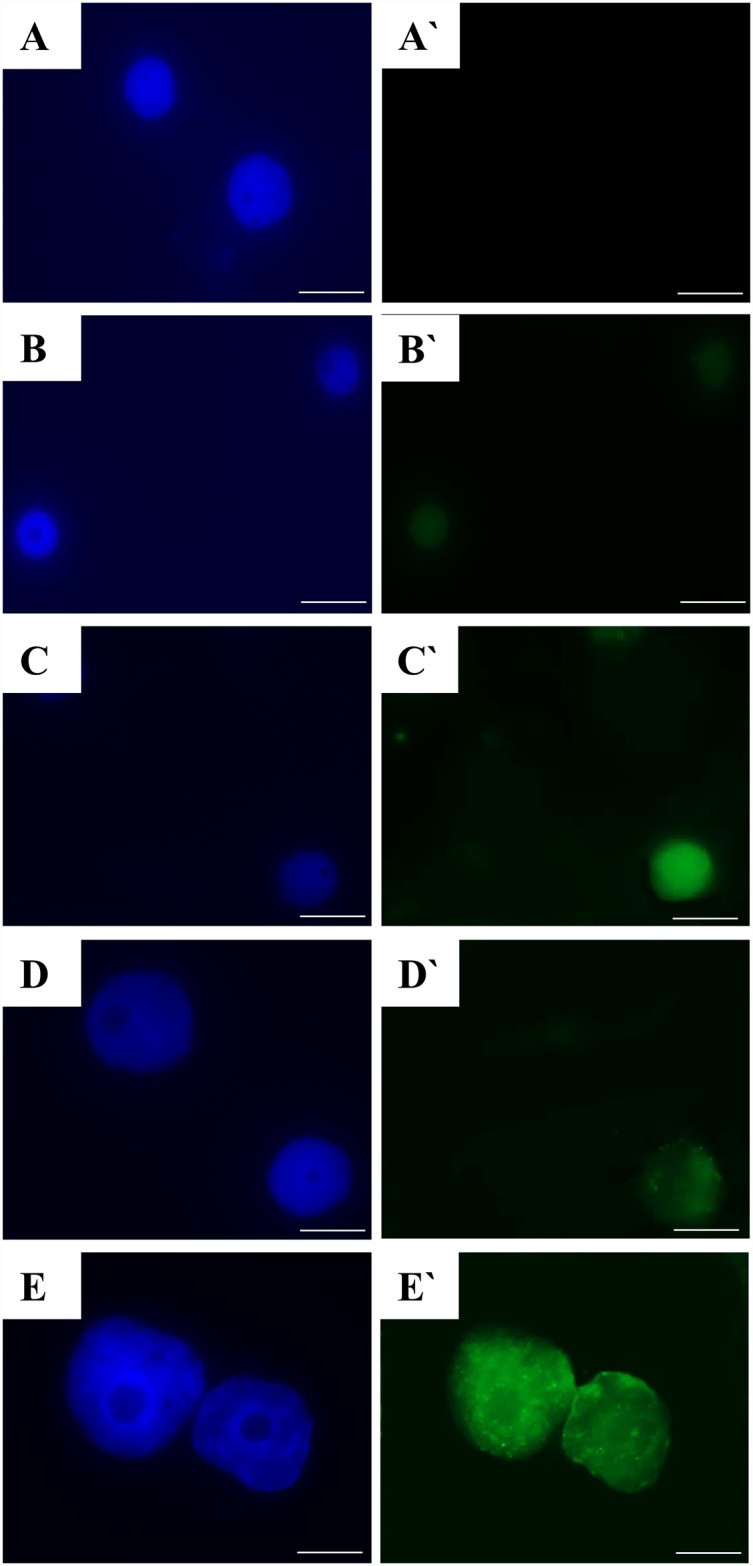
Examples of the *in situ* detection of DNA fragmentation using the TUNEL test in the MC and NC lines: 1–10 (A—A`), K5 (B—B`), 2–3 (C—C`), 10p^2^A (D—D`) and 6p1 (E—E`). Blue fluorescence: DAPI—all nuclei stained (A-D); green fluorescence: fluorescein-positive results of TUNEL test (A`-D`). Scale bars: 10 μm.

The application consisting of the TUNEL test permitted us to visualise the nuclei with DNA fragmentation in both the MC and NC lines of *F*. *tataricum*. Simultaneously with the detection of nuclei with DNA fragmentation (green fluorescence; Fig 7A–7E), DAPI staining was applied in order to show both TUNEL-positive and TUNEL-negative nuclei (blue fluorescence; Fig 7A–7E). When a whole callus tissue was sampled, TUNEL-positive nuclei were detected in both the MC (K5 and 2–3) and NC (10p^2^A and 6p1) callus lines (Fig 7). Interestingly, no DNA damage was revealed in the TUNEL test in lines 1–10 of the morphogenic callus. Almost all of the nuclei showed a green fluorescence of the FITC in the positive control in which a DNase solution was used to induce DNA strand breaks and no positively labelled nuclei were observed in the negative control of the TUNEL reaction (S1A, S1A`, S1B and S1B` Fig).

**Fig 8:**
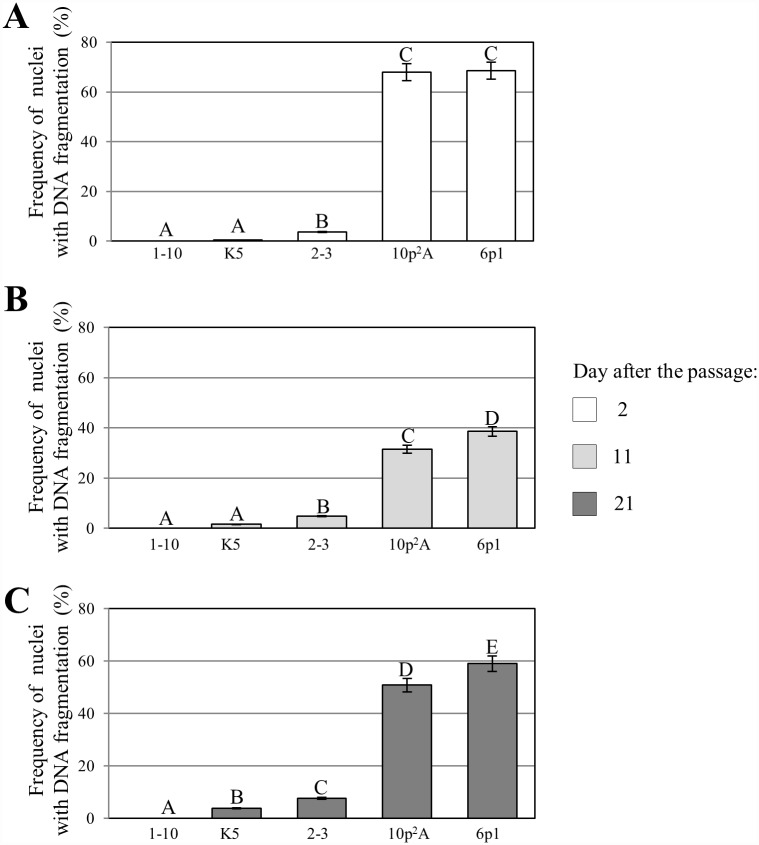
The frequencies of TUNEL-positive nuclei in all of the studied callus lines 2 (A), 11 (B) and 21 (C) days after the passage. Values with different letters differ significantly for the Student's t-test at P < 0.05.

**Fig 9:**
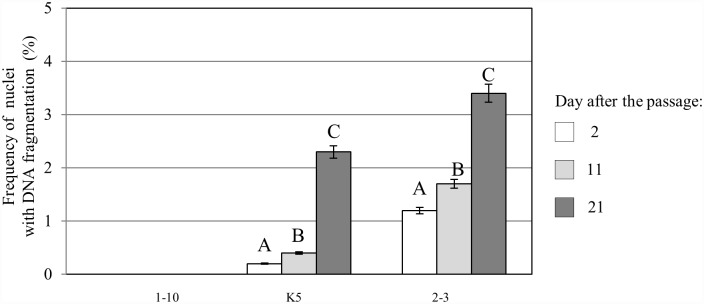
The frequencies of TUNEL-positive nuclei in the PECCs that originated from the callus lines 1–10, K5 and 2–3 2, 11 and 21 days after the passage. Values with different letters within the same callus line differ significantly for the Student's t-test at P < 0.05.

The results of the TUNEL test confirmed that the frequencies of the labelled nuclei were different for the different callus lines used in this study (Fig 8A–8C). We revealed significantly higher frequencies of positively labelled nuclei in the NC lines than in the MC lines. In the case of the morphogenic callus, the highest observed frequency of TUNEL-positive was 7.7% for lines 2–3. The lowest frequency of DNA-damaged nuclei in the NC was 31.5% in line 10p^2^A, whereas the highest level of DNA damage (68.5%) was observed in line 6p1. These results clearly indicate greater genome stability in the MC lines. A correlation between the frequency of TUNEL-positive nuclei and the length of time after the passage onto a fresh medium of the analysis was recorded for all of the callus lines with DNA damage. The highest frequencies of damaged nuclei were observed for the NC callus lines on the 2^nd^ day after the passage. Then, a decrease in the frequency of TUNEL-specific nuclei was observed on the 11^th^ day after the passage, while the observations that were carried out on the 21^st^ day after the passage showed a subsequent re-increase of the frequency of DNA-damaged nuclei, although not to the levels observed after the 2^nd^ day after the passage. The frequency of DNA-damaged nuclei in the MC callus, except for lines 1–10, gradually increased from the 2^nd^ to the 21^st^ day of the culture. Simultaneous with the TUNEL test that was applied to the whole callus samples, the PECCs from the MC lines were isolated and used for a TUNEL reaction. The frequencies of DNA-damaged nuclei in these cells were lower than in the whole callus sample of the corresponding lines (Fig 9). The highest frequency of DNA-damaged nuclei in the PECC cells (3.4%) was recorded for lines 2–3, whereas the corresponding value for the whole callus sample was 7.7%. This finding clearly demonstrates the greater stability of the PECC cells compared with the MC as a whole. The accumulation of DNA damage appears to be connected with senescence. An increase in single-strand-preferring nuclease activity was observed during dark-induced senescence in barley as well as during the natural senescence of wheat and rye leaves [61]. The main contribution to the appearance of DNA-damaged nuclei in the MC was apparently made by the senescent cells of the ‘soft’ callus and the senescent phenolic-containing surficial cells of the PECCs. These cells have the senescent-like features of the nuclei, which were flat and had chromatin condensation. The part of the ‘soft’ callus dominated the PECC part in the senescing cultures. Changes in the frequency of damaged nuclei during a subculture were previously demonstrated for the *Crepis capillaris* callus [62], where those authors revealed that the level of DNA damage decreased after the tissue was transferred onto a new medium but increased up to the 12^th^ day of culture. A similar response, which was observed for the PECC cells of Tartary buckwheat, can be explained by the exhaustion of nutrients, as well as the accumulation of metabolic products in the medium. The different response appears to be connected with another physiological and genetic status of the NC. One of the most important reasons for the nucleus damage appears to be connected with the high level of oxidative stress, which is a normal physiological state for the NC and its cells appear to be adapted to it. As was demonstrated earlier [21], the NC cells of *F*. *tataricum* are in a state of continuous oxidative stress, which is reflected by their higher content of hydrogen peroxide and malonic dialdehyde, low catalase activity and high activity of superoxide dismutase compared to the MC cells. It should be noted that high levels of lipid peroxidation and the malondialdehyde accumulation are typical for a fully habituated non-organogenic sugar beet callus [63]. Although the TUNEL test has previously been applied in some analyses of DNA damage in callus cells [6, 62, 64], our study is still quite a rare example of this type of application.

To conclude, in the subsequent research, we found that the callus lines of buckwheat are a remarkable example of strikingly different levels of genetic stability and either the analysis of chromosome number or preferably a detailed analysis of their rearrangements using fluorescence *in situ* hybridisation may enable a more complete characterisation of those callus lines. The constant monitoring of the morphology and regeneration capabilities of lines K5, 1–10 and 2–3 during a prolonged *in vitro* culture seems to be equally important.

## Supporting information

S1 Fig *In situ* detection of DNA fragmentation in the callus line K5 using the TUNEL test. (A—A`) positive control, (B—B`) negative control. Blue fluorescence: DAPI—all nuclei stained (A-B); green fluorescence: fluorescein-positive results of TUNEL test (A`–B`). Scale bars: 10 μm.
(TIF)
