# Supplementary figures and images for: Nuclear genome stability in long-term cultivated callus lines of Fagopyrum tataricum (L.) Gaertn
Source: PLoS One. 2017 Mar 9;12(3):e0173537. doi: 10.1371/journal.pone.0173537 (PMC5344457; doi:10.1371/journal.pone.0173537)

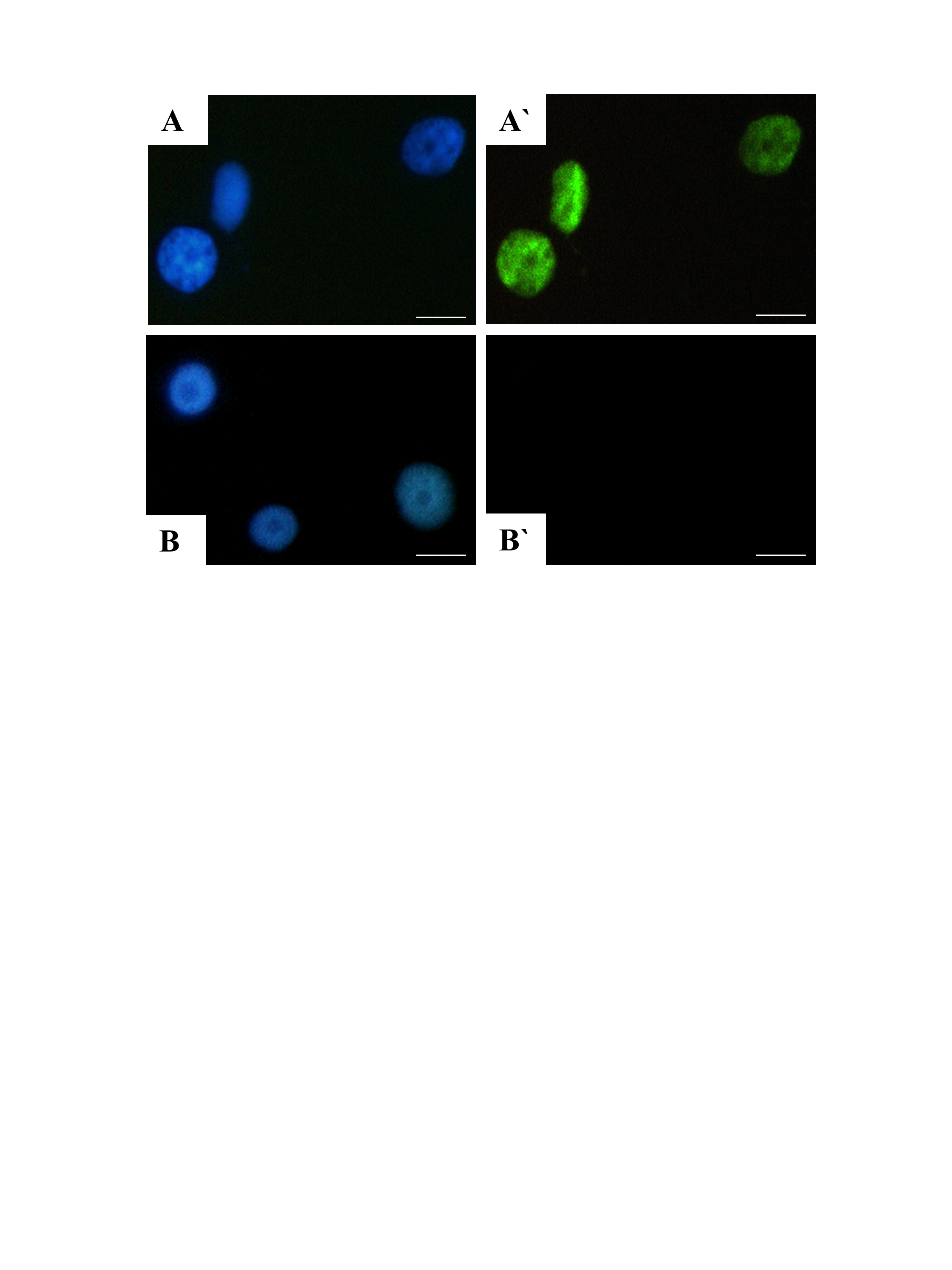

Supplement: S1 Fig — (A—A`) positive control, (B—B`) negative control. Blue fluorescence: DAPI—all nuclei stained (A-B); green fluorescence: fluorescein-positive results of TUNEL test (A`–B`). Scale bars: 10 μm. (TIF) [file pone.0173537.s001.tif]
